# Insights into Kinetics of Agitation-Induced Aggregation of Hen Lysozyme under Heat and Acidic Conditions from Various Spectroscopic Methods

**DOI:** 10.1371/journal.pone.0142095

**Published:** 2015-11-16

**Authors:** Ali Chaari, Christine Fahy, Alexandre Chevillot-Biraud, Mohamed Rholam

**Affiliations:** 1 ITODYS, UMR CNRS 7086, Univ. Paris Diderot, Sorbonne Paris Cité, 75205, Paris, France; 2 Laboratoire de Génétique et Biologie Cellulaire, Université de Versailles Saint-Quentin-en-Yvelines, 78035, Versailles, France; Aligarh Muslim University, INDIA

## Abstract

Protein misfolding and amyloid formation are an underlying pathological hallmark in a number of prevalent diseases of protein aggregation ranging from Alzheimer’s and Parkinson’s diseases to systemic lysozyme amyloidosis. In this context, we have used complementary spectroscopic methods to undertake a systematic study of the self-assembly of hen egg-white lysozyme under agitation during a prolonged heating in acidic pH. The kinetics of lysozyme aggregation, monitored by Thioflavin T fluorescence, dynamic light scattering and the quenching of tryptophan fluorescence by acrylamide, is described by a sigmoid curve typical of a nucleation-dependent polymerization process. Nevertheless, we observe significant differences between the values deduced for the kinetic parameters (lag time and aggregation rate). The fibrillation process of lysozyme, as assessed by the attenuated total reflection-Fourier transform infrared spectroscopy, is accompanied by an increase in the β-sheet conformation at the expense of the α-helical conformation but the time-dependent variation of the content of these secondary structures does not evolve as a gradual transition. Moreover, the tryptophan fluorescence-monitored kinetics of lysozyme aggregation is described by three phases in which the temporal decrease of the tryptophan fluorescence quantum yield is of quasilinear nature. Finally, the generated lysozyme fibrils exhibit a typical amyloid morphology with various lengths (observed by atomic force microscopy) and contain exclusively the full-length protein (analyzed by highly performance liquid chromatography). Compared to the data obtained by other groups for the formation of lysozyme fibrils in acidic pH without agitation, this work provides new insights into the structural changes (local, secondary, oligomeric/fibrillar structures) undergone by the lysozyme during the agitation-induced formation of fibrils.

## Introduction

Cellular systems maintain the balance between protein synthesis and degradation via the quality-control machinery that prevents deposition of partially folded, misfolded or degraded protein in the cells [1]. Conversely, a deregulation of these systems (*i*.*e*. change in cellular environment, genetic mutations, aging) can lead to various human diseases such as neurological disorders (*i*.*e*. Alzheimer's and Parkinson's diseases) and various systematic amyloidosis [2]. These degenerative diseases are characterized by the formation and deposition of amyloid fibrils in different tissues and organs, resulting from uncontrolled self-aggregation of unfolded proteins [3–5]. However, living organisms also exploited the formation of amyloid fibrils as mechanism to perform physiological functions in specific biological contexts [6,7]. For example, these amyloid fibrils can act as functional bacterial coatings [8], natural protective systems against predators for various species [9], catalytic template in mammalian skin pigmentation [10] or as natural storage for peptides and proteins in secretory vesicles [11]. In these cases, the formation of such protein amyloids is tightly controlled and regulated by cells whereas the aggregation of proteins in the degenerative diseases refers to their abnormal self-association.

Besides these observations, it was also shown that all the proteins and peptides are able to form *in vitro* amyloid aggregates [12–20], thus indicating that the formation of amyloid fibrils is an intrinsic property of polypeptide chains [21]. By taking advantage of this generic property of proteins, investigation of amyloid fibrillation using non-disease-associated proteins such as hen egg white lysozyme (HEWL) can help in deciphering the molecular mechanisms of amyloid fibrillogenesis. HEWL is an archetypal protein widely used to study the mechanisms of protein folding, misfolding and amyloid formation [22,23]. The native structure of this protein is composed of two different domains (α and β) cross-linked by four disulfide bonds [24]. The α-domain is constituted by four α-helices whereas the β-domain consists mainly of an antiparallel β-sheet. Both domains are functional for the active site cleft which is formed between them. Additionally, HEWL is a multitryptophan-containing protein which possess six Trp residues distributed throughout its tertiary structure: four situated in the α-domain (Trp^28^, Trp^108^, Trp^111^ and Trp^123^) and two in the β-domain (Trp^62^ and Trp^63^). Furthermore, HEWL is structurally homologous to the human lysozyme whose familial mutations are associated with lysozyme systemic amyloidosis [23–26]. Finally, lysozyme is able to form fibrils under various *in vitro* conditions [27–43] and it has been reported that HEWL aggregates are toxic to cell cultures [32].

In the present paper, we attempt to elucidate the mechanism of HEWL aggregation by characterizing the molecular features of aggregate species that form along the process by which HEWL assembles into amyloid fibrils under heat and acidic conditions with agitation. To achieve this goal, the aggregation kinetics of HEWL has been monitored by using various and complementary spectroscopic techniques. Hence, the formation of amyloid aggregates was monitored by means of the Thioflavin T (ThT) fluorescence and the morphology of such protein aggregates was analyzed by the atomic force microscopy (AFM). The distribution of protein species, formed during the aggregation process, and their size growth were investigated by the dynamic light scattering (DLS). Changes at the secondary structure level of HEWL aggregates were followed by means of the attenuated total reflectance (ATR)-Fourier transform infrared spectroscopy (FTIR). Given that HEWL is a multitryptophan-containing protein, the fluorescence characteristics of its Trp residues were used to probe the changes occurring in the tertiary conformation of oligomeric species formed during the protein aggregation process. Finally, because a prolonged heating in acidic pH may cause covalent changes in the protein, the integrity of lysozyme within the oligomeric/fibrillar structures was checked by the reverse phase (RP)-highly performance liquid chromatography (HPLC) technique.

## Materials and Methods

### Materials

Hen egg-white lysosyme (EC 3.2.1.17) and thioflavin T were purchased from Sigma-Aldrich (St. Louis, MO). Tris(2-carboxyethyl) phosphine (TCEP) was purchased from Cayman Chemical Company (Michigan, USA). All other reagents and buffer components were of analytical grade.

### Lysozyme aggregation

The sample solutions of Hen egg white lysozyme (HEWL), without further purification, were prepared in 10 mM glycine buffer (pH 2.0) containing 0.2% (w/v) sodium azide. The concentrations of HEWL were determined spectrophotometrically at λ = 280 nm using molar extinction coefficient of 37,970 M^−1^cm^−1^ [42]. To produce the amyloid structures, HEWL solutions (1.36 mM) were incubated for different days at 55°C in a thermomixer with agitation of 700 rpm. At regular time intervals, samples for analysis were taken and stored at 4°C. The sample solutions of Hen egg white lysozyme (HEWL), without further purification, were prepared in 10 mM glycine buffer (pH 2.0) containing 0.2% (w/v) sodium azide.

### Thioflavin T (ThT) fluorescence assay

To monitor the aggregation of HEWL, the fluorescent ThT dye was added to the protein samples (10 μM) to a final concentration of 20 μM. The concentration of ThT was determined spectrophotometrically at λ = 412 nm using molar extinction coefficient of 36,000 M^−1^cm^−1^ [42,43]. The fluorescence emission spectra of ThT were collected from 450 nm to 550 nm on a Bowman fluorescence spectrophotometer using an excitation wavelength of 440 nm [32]. Fluorescence measurements were performed at 25°C in 1 cm quartz cell with both excitation and emission bandwidths of 5 nm. The fluorescence spectra of HEWL samples were determined by subtracting the fluorescence of ThT blanks. Each experiment was performed in quadruplicate. The values of the quantum yield of ThT fluorescence were determined by integrating the emission spectra from 450 nm to 500 nm.

### Atomic Force Microscopy (AFM) measurement

AFM images were acquired in non-contact mode in a vibration-insulated environment using a PicoPlus microscope (Molecular Imaging) equipped with a NanoScan-3000 controller. For imaging, we used single beam aluminium-coated cantilevers (type NSC36/ALBS, μmasch) with Rc<10 nm, 110–130 μm lengths and nominal spring constant (0.6 N/m). The drive frequency was between 200 and 400 kHz. The HEWL solutions were diluted 400 times and a small aliquot (20 μl) was deposited on freshly cleaved mica. The samples were incubated on mica for 10 min followed by three washes with 50 μl water to gently remove the material not adsorbed to the substrate. The sample was dried under mild vacuum and imaged in air. Each experiment was performed in quadruplicate at 25°C. The acquisition and the analysis of AFM pictures were performed by using the softwares “Nanoscope 5.30r3sr3” and “WSxM 5.0 Develop 3.1”, respectively.

### Dynamic Light Scattering (DLS) measurement

DLS measurements were carried out using a DynaPro MS800 instrument (Wyatt Technologies Corp.) equipped with a gallium aluminium arsenide 825 nm laser. The total light scattering intensity of HEWL solutions (1 mg/ml) was collected at a 90° angle. All measurements were made in 3 mm quartz cell at 25°C. The acquisition of data (usually 30–40 points) was made with an acquisition time of 30 s and the obtained data were averaged. The autocorrelation curves were deconvoluted using Dynamics V6 software to obtain size distribution and hydrodynamic radii (<R_h_>). Each experiment was repeated three times to ensure reproducibility.

### Fourier Transform Infra Red spectroscopy (FTIR) analysis

Attenuated total reflectance Fourier transform infrared (ATR-FTIR) spectra of HEWL samples were recorded on a FTIR spectrometer (model IFS-66v; Nicolett) equipped with a horizontal ZnS ATR accessory. 50 μl of samples (1 mg/ml) were placed directly in the ZnS ATR accessory and the spectra were recorded at 25°C. 200 scans were performed for each spectrum at 2 cm^−1^ resolution. The buffer background, independently measured, was subtracted from the protein spectra before curve fitting of the amide I region. Each experiment was repeated three times to ensure reproducibility. To identify the different spectral components of HEWL species and to determine their respective content, the spectra were analyzed by using the Grams 31 program version 4.14 (Galactic Industries Corporation, Salem, NH).

### Intrinsic fluorescence assay

The fluorescence emission spectra (collected from 300 nm to 500 nm) of 20-fold diluted lysozyme samples were acquired with a Bowman fluorescence spectrophotometer. The excitation wavelength was set at 295 nm in order to observe exclusively the fluorescence of Trp residues. Fluorescence measurements were performed at 25°C in 1 cm quartz cell with both excitation and emission bandwidths of 2 nm. The fluorescence spectra of protein samples were determined by subtracting the fluorescence of buffer and corrected for scattering effect [44]. Each experiment was performed in quadruplicate. The values of the quantum yield of Trp fluorescence were determined by integrating the fluorescence emission spectra from 310 nm to 450 nm.

### Fluorescence quenching study

Quenching titrations with acrylamide were carried out by addition of varying amounts of a quencher stock solution (5 M) to the protein solution (~10.0 μM). The excitation wavelength was set at 295 nm and the fluorescence emission spectra of Trp (collected from 300 nm to 500 nm) were acquired on a Bowman fluorescence spectrophotometer. Fluorescence measurements were performed at 25°C in 1 cm quartz cell with both excitation and emission bandwidths of 2 nm. After subtracting the fluorescence spectra of buffer containing the acrylamide, the fluorescence emission spectra were corrected for volume changes, scattering effect and the inner filter effect due to acrylamide absorption [44]. Each experiment was performed in quadruplicate. The values of the quantum yield of Trp fluorescence were determined by integrating the fluorescence emission spectra from 310 nm to 450.

### Reverse phase (RP)-highly performance liquid chromatography (HPLC) analysis

To check the integrity of lysozyme within the oligomeric/fibrillar structures, the HPLC experiments were performed on a Waters high-performance liquid chromatography system equipped with a solvent delivery system, a diode-array UV-Vis detector and an autosampler. HEWL samples, obtained at regular time intervals, were firstly exposed to 5 M guanidium chloride (Gnd-HCl) solution for a time period of 24 h in order to dissociate the oligomeric/fibrillar species of the protein. Secondly, the reducing agent Tris(2-carboxyethyl) phosphine (TCEP) was added to the resulting solutions of HEWL ([TCEP/HEWL]≈100) and incubated at room temperature for different lengths of time in order to reduce the disulfide bonds of HEWL. The chromatographic separation of each HEWL solution was carried out using a Vydac C18 column (150 x 4.6 mm) eluted with a linear gradient of acetonitrile/0.1% TFA from 5% to 20% in five minutes, from 20% to 50% in 15 minutes and from 50% to 70% ten minutes. Elution was performed at a flow rate of 1.0 ml/min and absorbance of samples was monitored at 215 and 280 nm.

### Analysis of fibril formation kinetics

All aggregation curves were fitted to a sigmoidal function [45], implemented within the Origin 6.0 software package (Microcal, Southampton, MA), to extract the relevant aggregation parameters (see Eq 1).

$$S=[S_{i}\;-\;S_{f}]/[1+e^{(t-t1/2)/\tau }]$$ (1)

where S is the observed signal, t is the time, S_i_ and S_f_ are the initial and final values of the signal, respectively. The values of the parameters of the sigmoidal curve t_1/2_ (half-time: time required to reach the half of the maximum of the signal S_f_) and τ (magnitude of the signal change) were determined by fitting the experimental data.

## Results

### Kinetics of HEWL oligomerization process monitored by Thioflavin T (ThT) binding

**Fig 1:**
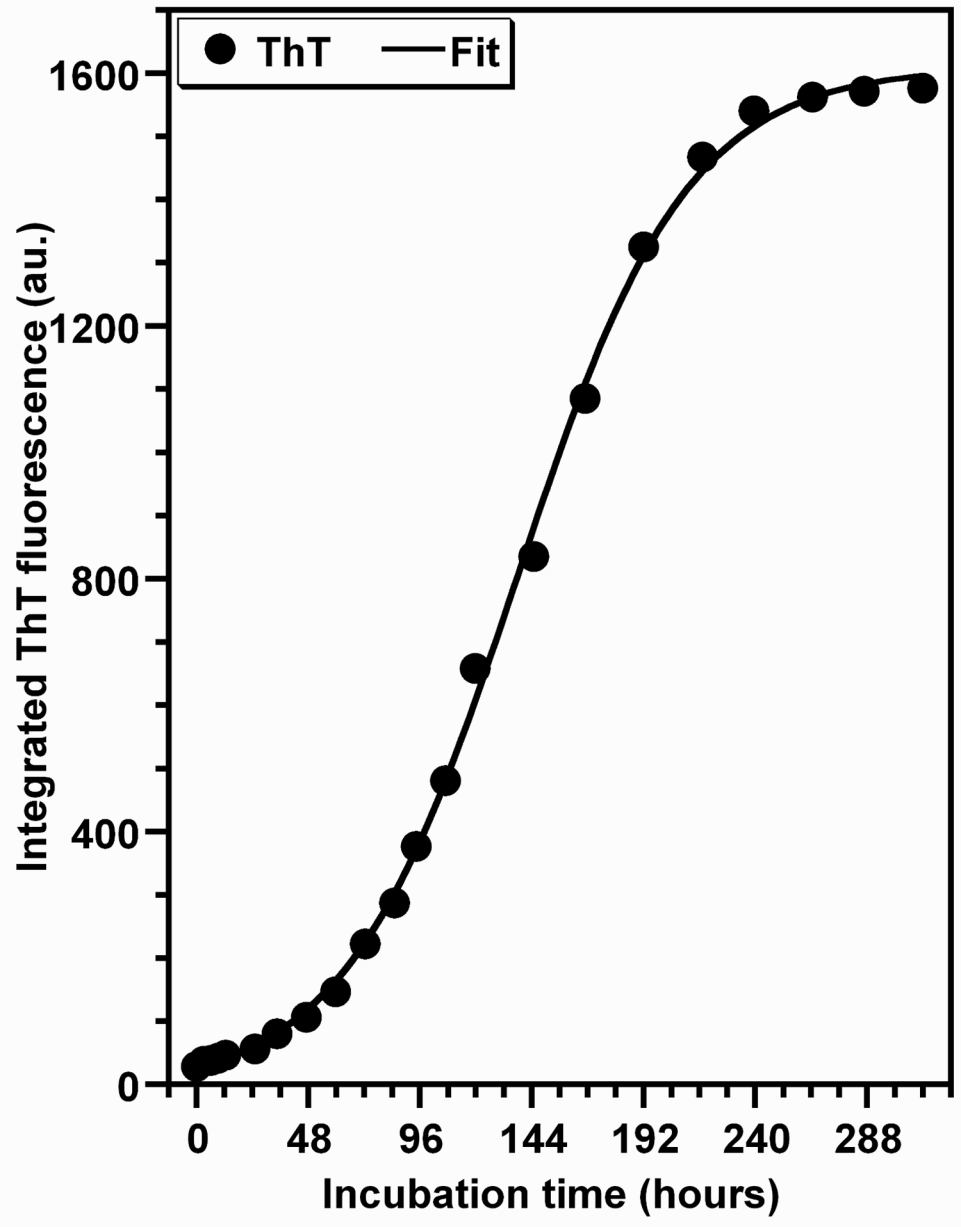
Amyloid fibrillogenesis kinetics of HEWL followed by ThT fluorescence. The data represent the temporal evolution of the quantum yield of ThT fluorescence during the aggregation process of HEWL. The values of the fluorescence quantum yield of ThT are the mean of three independent measurements, each performed in quadruplicate. HEWL solutions were subjected to this analysis at the concentration of 10 μM. The line of best fit through the data points was obtained by fitting the data with a sigmoidal function [45].

To monitor the kinetics of HEWL aggregation, we undertook *in vitro* aggregation measurements in the presence of the ThT dye; the fluorescent properties of which change upon its binding to amyloid fibrils of proteins [46]. Fig 1 shows the time-dependent enhancement of the quantum yield of ThT fluorescence during the oligomerization process of HEWL under our experimental conditions (pH = 2.0, T = 55°C and agitation = 700 rpm). The ThT fluorescence curve exhibits a shape characteristic of a sigmoid curve generally interpreted as the consequence of a nucleation-dependent polymerization process with a lag phase, a growth phase and an equilibrium phase [21,45]. From the fitted kinetic data to a sigmoidal function [45], the obtained values of the half-time for aggregation (t_1/2_) and the apparent rate constant (k_app_ = 1/τ) are 138.0 h and 0.028 h^-1^, respectively. The lag time (t_lag_[ThT] = t_1/2_−2τ), deduced from these parameters, has a value of 66.6 h. Generally, these parameters are dependent of several factors such as the protein concentration, temperature, pH and cosolvents [27–41].

### Morphology of HEWL aggregates observed by Atomic Force Microscopy (AFM)

**Fig 2:**
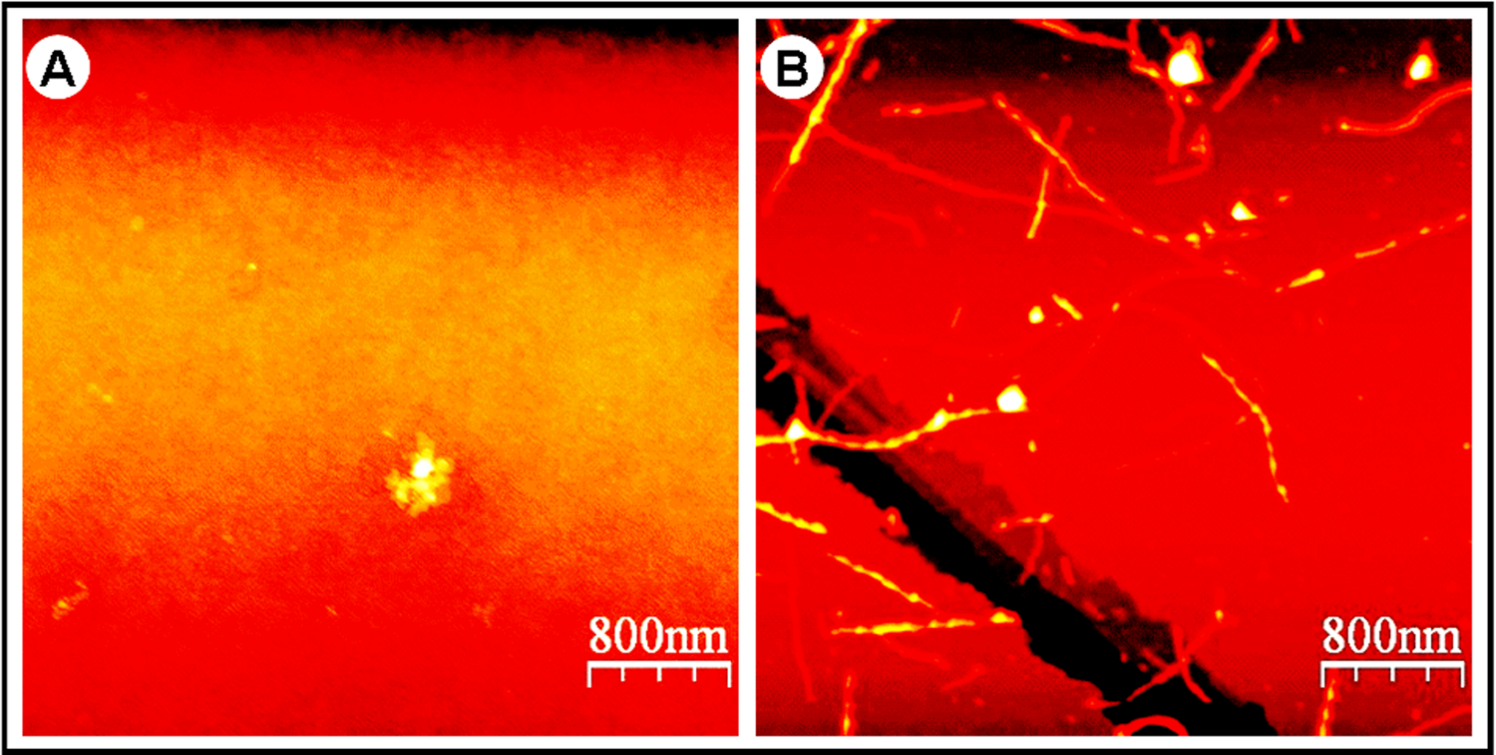
AFM images of HEWL aggregates. AFM images of an aliquot from the incubation solution were obtained at 3 h time-point in the lag phase (A) and at 240 h time-point in the equilibrium phase (B). AFM images of HEWL solutions were registered at the concentration of 10 μM.

Given that non-fibrillar aggregates of proteins may also show high ThT fluorescence signal, the formation of HEWL amyloids was confirmed by investigating the morphology of HEWL aggregates using atomic force microscopy (AFM). Compared to the AFM image of HEWL obtained at 3 h time-point in the lag phase (Fig 2A), the AFM image of HEWL obtained after 240 h of incubation (Fig 2B) shows the presence of fibrillar aggregates, thus confirming the observed increase in the ThT quantum yield (Fig 1) in agreement with several reports [27–41]. The analysis of this AFM image by the software “WSxM 5.0 Develop 3.1” (see materials and methods) reveals heterogeneity in the size of fibrillar species of HEWL. These fibrillar aggregates were classified according to their lengths (L): short fibrils (70 nm ≤ L < 200 nm, percentage = 38%), intermediate fibrils (200 nm ≤ L ≤ 500 nm, percentage = 39%) and long fibrils (L > 500 nm, percentage = 23%).

### Size of growing HEWL oligomers as assessed by Dynamic Light Scattering (DLS)

DLS spectroscopy has proven to be a powerful method for monitoring the growth of oligomeric particles upon aggregation as well as for determining the size distribution of protein assemblies [47].

**Fig 3:**
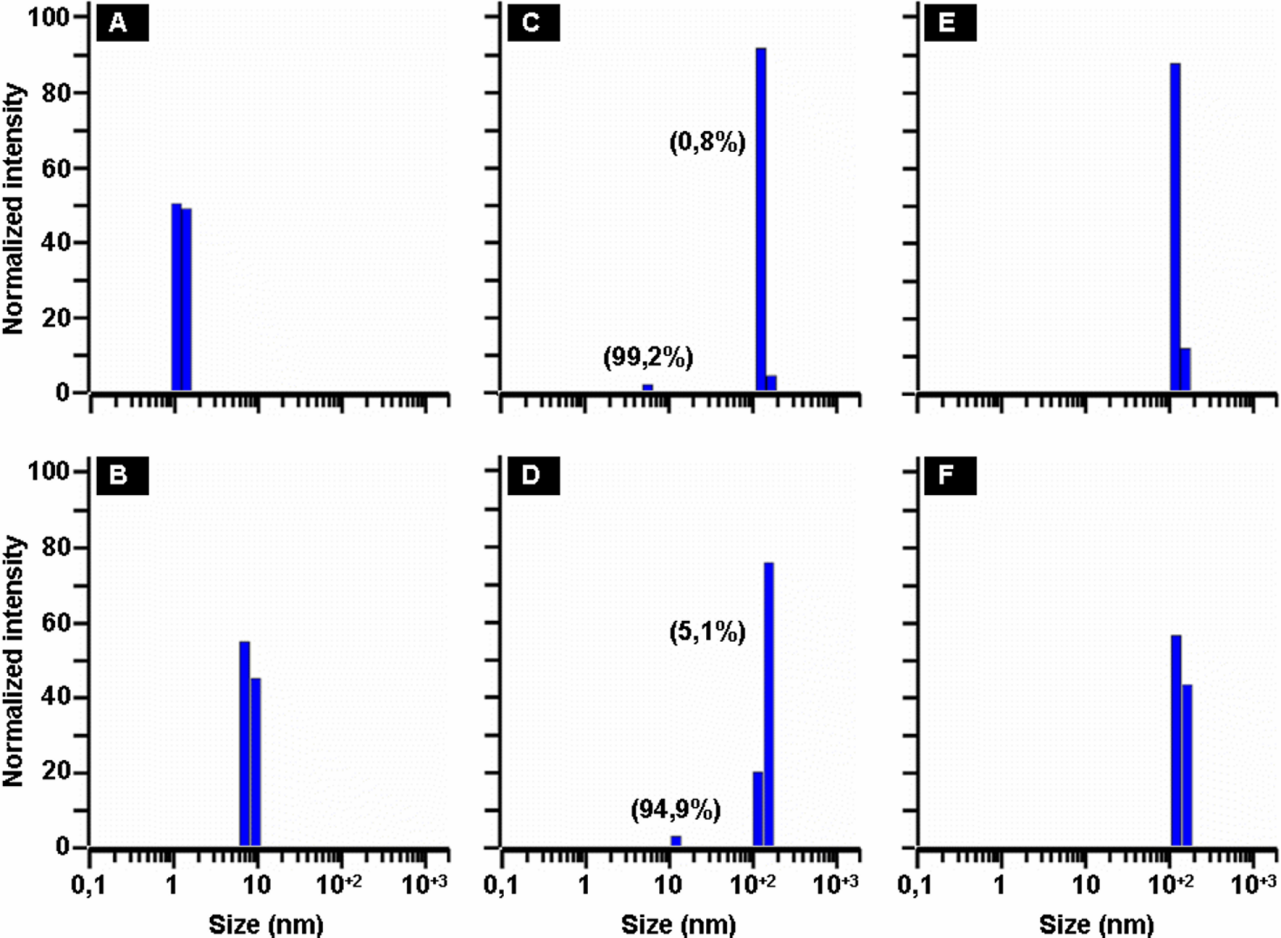
Size distribution of HEWL assembly revealed by DLS. The panels represent the DLS graphs of size distribution of HEWL particles estimated from the intensity percentage at 0 h (A), 48 h (B), 72 h (C), 96 h (D), 168 h (E) and 240 h (F). The values of the percentage of particles in mass (in parenthesis) are the mean of three independent measurements, each performed in triplicate. Measurements of HEWL solutions were done at the protein concentration of 1 mg/ml.

Fig 3 shows the representative DLS graphs of size distribution of HEWL particles estimated from the intensity percentage at different incubation times. Analysis of these graphs shows that the distribution of protein aggregates with their size remained unimodal and the polydispersity index of each peak does not exceed 14%. The value of the initial average hydrodynamic radius (<R_h_>) of particles is around 1.2 nm (Fig 3A), which agrees with the radius for predominantly monomeric lysozyme [48]. When the incubation time increases, the hydrodynamic radius of HEWL particles grows to reach a value of 52.0 nm for an incubation time of 240 h (a time corresponding to the stationary phase) (Fig 3I). Moreover, the DLS graphs of HEWL particles, obtained at the incubation times of 72 h (Fig 3D) and 96 h (Fig 3E), reveal the coexistence of two peaks corresponding to two populations of HEWL species.

**Fig 4:**
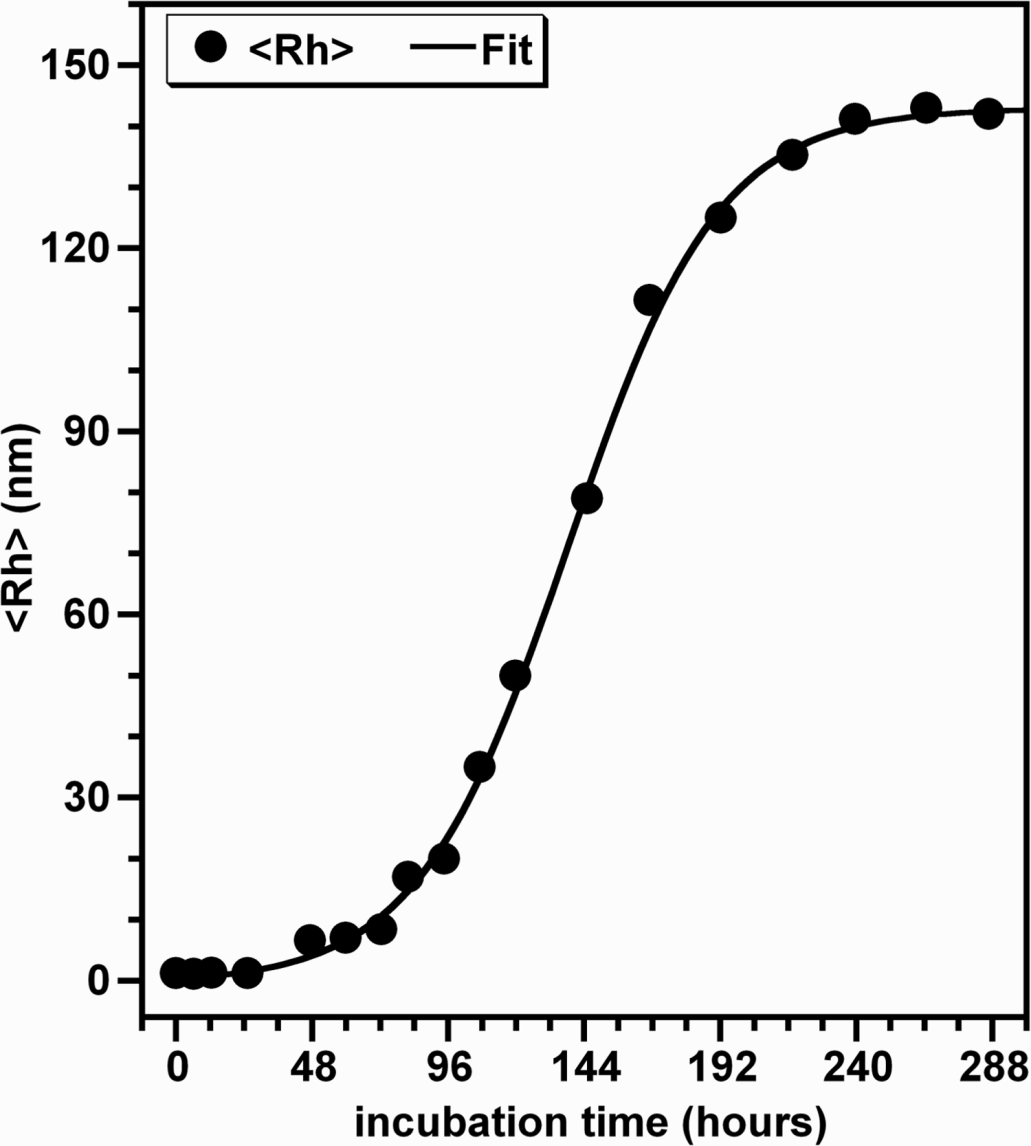
Oligomerization kinetics of HEWL monitored by DLS. The data represent the temporal evolution of the average hydrodynamic radius <*Rh*> of HEWL species formed during the aggregation process. The values of <Rh>, deduced from the data in the Fig 3, are the mean of three measurements, each performed in triplicate. The line of best fit through the data points was obtained by fitting the data with a sigmoidal function [45].

Fig 4 displays the temporal evolution of the hydrodynamic radius (<R_h_>) of the major population of HEWL species formed during the time course of the protein aggregation. The analysis of this variation on time shows that the experimental data are fitted reasonably well by a sigmoidal curve as shown for those obtained from the ThT fluorescence-monitored kinetics (Fig 1). The extracted values of t_1/2_ and k are 138.0 h and 0.039 h^-1^, respectively. The lag time, deduced from these parameters, has a value (t_lag_[DLS] = 86.7 h) which is higher than that obtained from the ThT fluorescence curve. This augmentation in t_lag_ arises essentially from the increase in the apparent growth rate constant. Interestingly, the increase in an exponential manner of the hydrodynamic radius with the incubation time was also observed for HEWL aggregation at alkaline pH but with no apparent lag phase [34].

### Secondary structures of HEWL aggregates as assessed by Fourier Transform Infrared (FTIR) spectroscopy

To gain insight into the conformation of protein assemblies formed under our experimental conditions, we have used the ATR-FTIR spectroscopy to monitor the changes in the secondary structures of HEWL along its fibrillation process. To this end, our analysis was focused on the amide I region (1600–1700 cm^-1^) that is due almost entirely to the C = O stretch vibrations of the peptide linkages [49].

**Fig 5:**
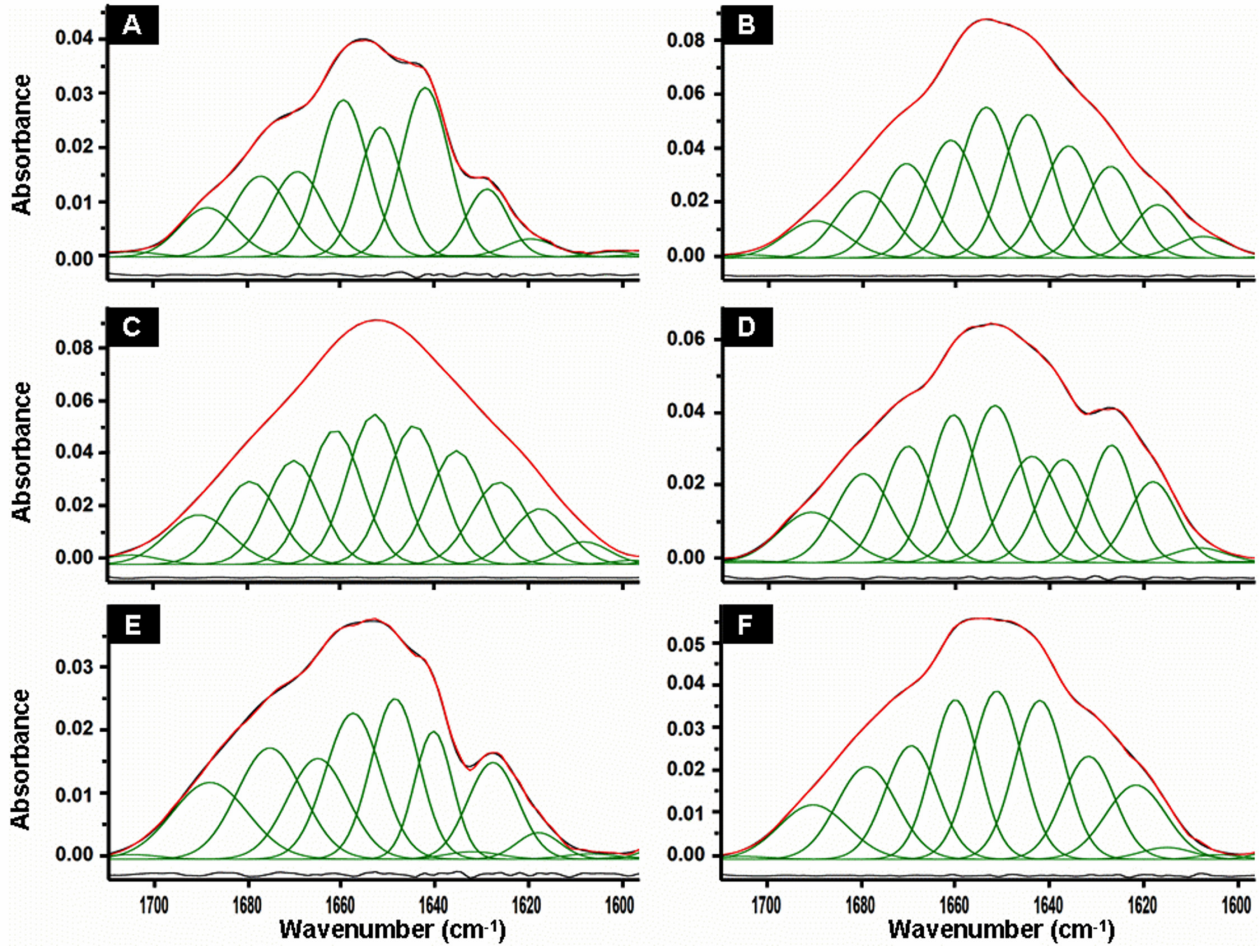
Infrared spectra of HEWL species recorded during the time course of the aggregation. The panels represent the ATR-FTIR spectra of HEWL solutions registered at 0 h (A), 48 h (B), 72 h (C), 96 h (D), 168 h (E) and 240 h (F). The spectra in green represent the individual secondary structure components of the protein identified by the treatment of original spectra by deconvolution and curve-fitting procedures [50,51]. Peaks in the regions 1616–1621 cm^-1^, 1626–1640 cm^-1^ and 1680–1694 cm^-1^ are mostly from ß-sheet, those around 1645 cm^-1^ from disordered structures, those centred between 1663 cm^-1^ and 1673 cm^-1^ from turns and loops, and from 1649 cm^-1^ to 1660 cm^-1^ generally from α-helix structure [50,51]. Measurements, obtained from three independent experiments, were performed for different protein concentrations.

**Fig 6:**
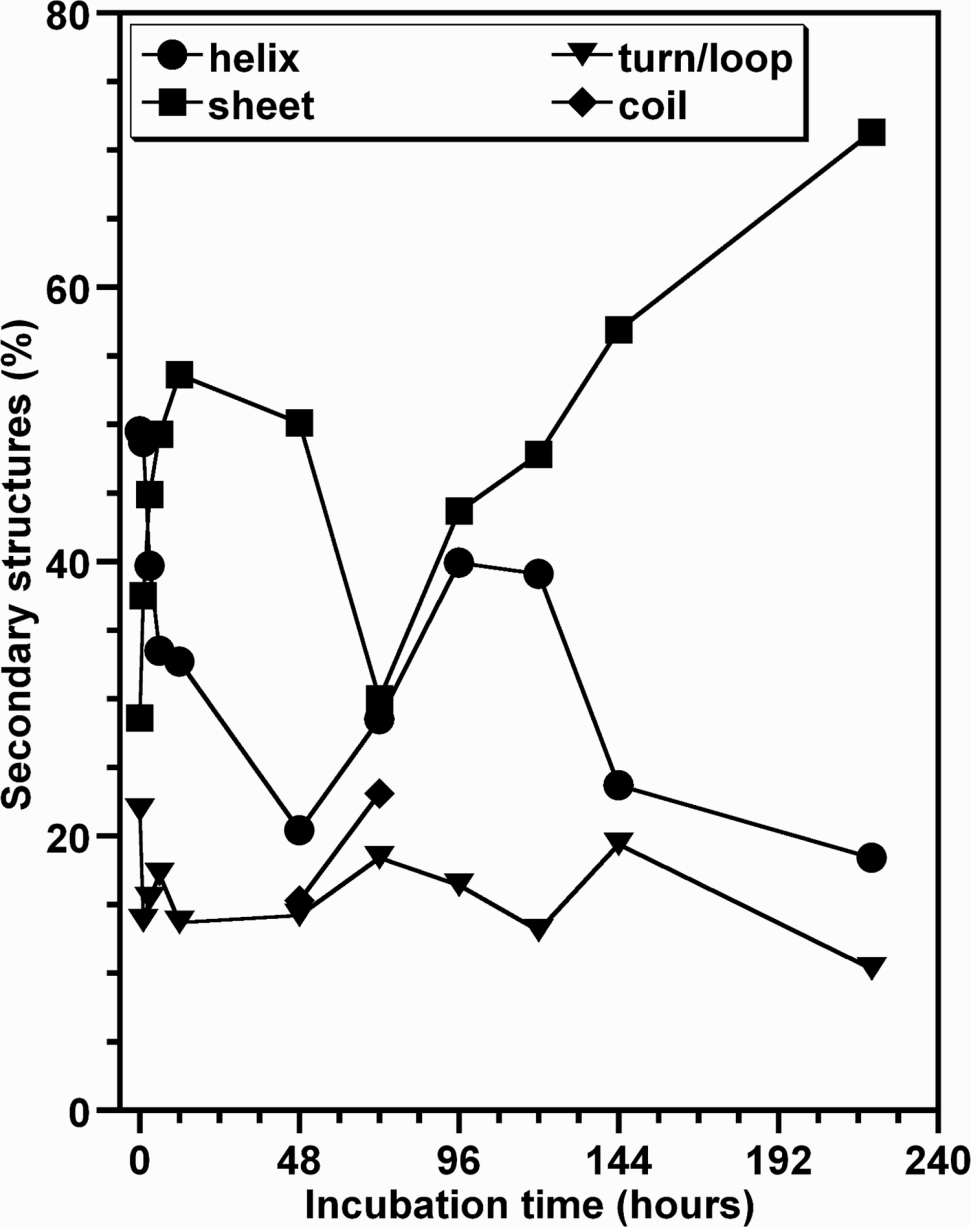
Aggregation kinetics of HEWL monitored by FTIR. The data represent the temporal evolution of the secondary structure contents of HEWL species determined by curve fitting of the ATR-FTIR spectra shown in the Fig 5. The values of percentages of the individual secondary structure components of the protein are the mean of three measurements, each performed in triplicate.

Fig 5 shows the FTIR spectra of HEWL species obtained during the different phases of the protein aggregation process and the individual secondary structure components deduced from the treatment of spectra by deconvolution and curve-fitting procedures [50,51]. The FTIR spectrum of the monomeric protein (Fig 5A) is characterized by a largely α/β native structure with a prevalence of α-helical structure represented by the bands situated between 1649 and 1660 cm^−1^ and of β-sheet structures evidenced by the presence of three characteristic bands localized in the regions 1616–1621 cm^-1^, 1626–1640 cm^-1^ and 1680–1694 cm^-1^ [49–51]. The remaining components, located between 1663 cm^-1^ and 1673 cm^-1^, are generally associated to the turn/loop structures [49–51]. For the lysozyme oligomers formed after about 10 days of incubation, the FTIR spectrum (Fig 5F) shows substantial changes essentially in terms of the intensities of the bands. Based on the treatment of FTIR spectra, the α-helix, β-sheet and turn/loops structures account for 49.5%, 28.6% and 21.8% of the total structure of the monomeric HEWL, respectively (Fig 6). In contrast, the HEWL fibrils contained 18.4% of α-helix, 71.3% of β-sheet and 10.4% of turn/loops (Fig 6). These results, which are consistent with those obtained for human, hen and equine lysozyme by other groups [35,38,52], indicate that the lysozyme oligomers/fibrils contain significant amounts of β-sheet structures, a common feature of several amyloidogenic proteins [53].

Fig 6 displays the time-dependent evolution of the content of the secondary structures of HEWL species. The analysis of this kinetics curve allowed to deduce the following observations. Firstly, the percentage of turns/loops undergoes a very slight change during the different phases of the aggregation process of HEWL. Secondly, we observe the presence of random coil structures between 48 h (15.0%) and 72 h (25.0%). Thirdly, the percentage of β-sheet structures exhibits a substantial increase during the first 48 hours (~50% of the total β-sheet structures detected throughout the aggregation process of HEWL) followed by a decrease until the end of the lag phase (t_lag_[ThT] ~ 72 h) and then increases progressively up to the end of the aggregation process. Finally, the temporal evolution of the amount of α-helix is approximately the mirror image of that of the percentage of β-sheet conformation. Although the formation of HEWL fibrils is accompanied by the conversion of the α-helix to ß-sheet structures, this FTIR analysis shows that the temporal evolution of the content of both secondary structures does not occur as a gradual transition.

### HEWL self-assembly analyzed by steady-state fluorescence of tryptophan

Fluorescence spectroscopy is one of the most useful methods to elucidate local and global conformations of proteins as well as protein-protein interactions [44,54]. Among the six Trp residues of HEWL, Trp^108^ and Trp^62^ (situated in the α and β domains, respectively) are known to contribute for ~80% of the total protein fluorescence [55,56]. Given the aforesaid features, the changes in the fluorescence properties of these residues [57–59] were exploited to obtain topological information on the assembly of HEWL

**Fig 7:**
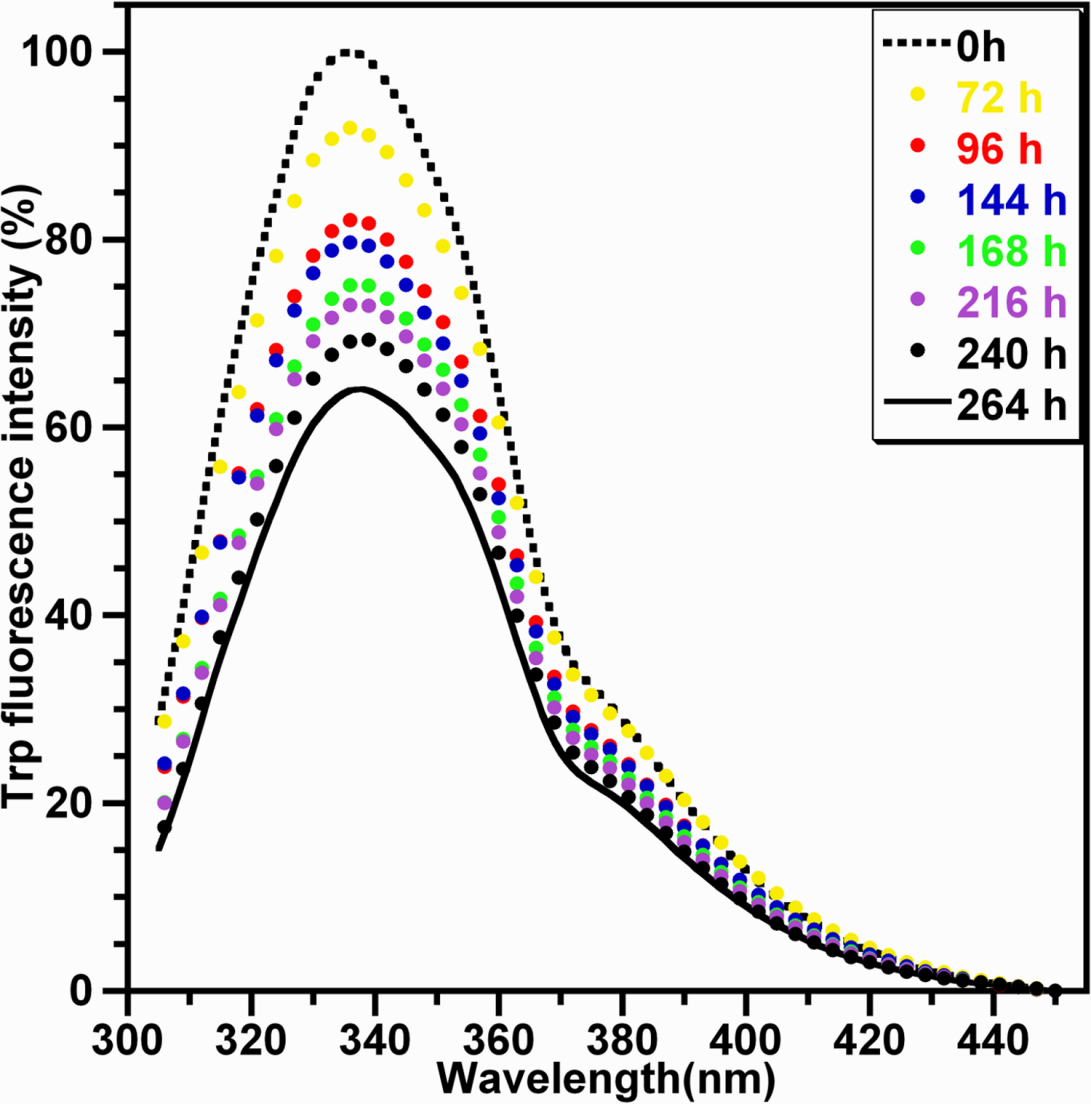
Trp fluorescence spectra of HEWL species recorded during the aggregation. The Trp emission spectra (excitation at 295 nm) of HEWL species, obtained at different incubation times, are the mean of four independent experiments. Fluorescence measurements of HEWL solutions were acquired at the protein concentration of 1 mg/ml.

Fig 7 reports on the Trp fluorescence emission spectra of HEWL species (excitation at 295 nm) obtained at different incubation times. As shown, the fluorescence spectrum of the monomeric HEWL (0 h) exhibit λ_max_ situated around 337±3 nm. Based on the values of λ_max_ observed for the Trp residues in the proteins [57], the Trp residues of HEWL can be considered as partially buried in the monomeric form at acidic pH (330 nm < λ_max_ < 345 nm). For the fibrillar HEWL (264 h), λ_max_ of its fluorescence spectrum undergoes a very slight red shift (Δλ_max_ ≤ 4 nm) whereas its fluorescence quantum yield is 2-fold lower than that of the HEWL monomers (0 h). Accordingly, the differences, observed between the Trp photophysical properties of the monomeric and oligomeric forms of HEWL, are indicative of the quenching of the Trp fluorescence (through different quenching mechanisms) by certain types of amino acid residues during the HEWL fibrillation [44,54,57,58]. These quenching interactions result probably from the intra- and/or inter-molecular interactions of the Trp microenvironment with neighbouring residues within the oligomeric/fibrillar forms of HEWL [55].

**Fig 8:**
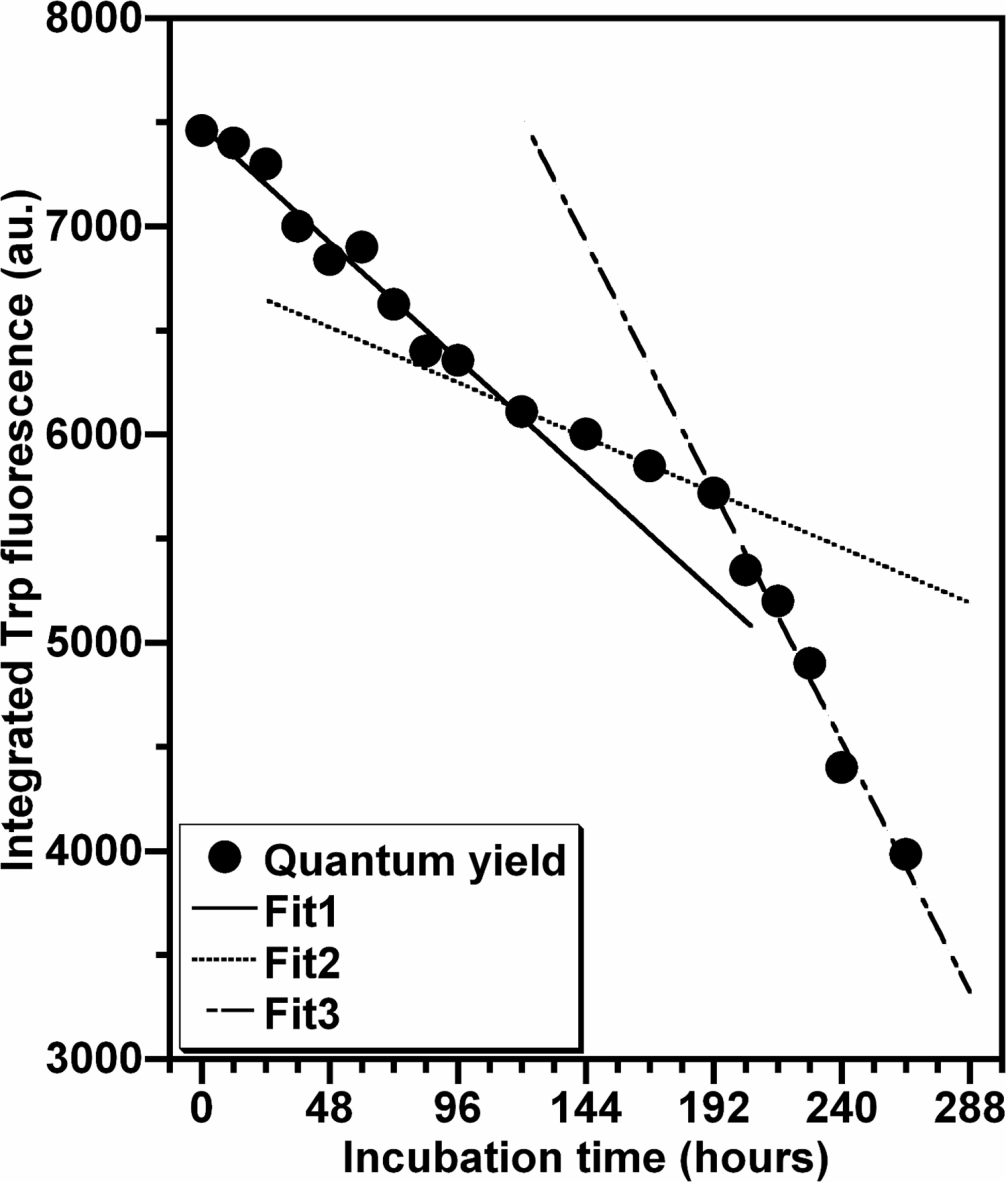
Aggregation kinetic of HEWL monitored by Trp fluorescence. The data represent the temporal evolution of the Trp fluorescence quantum yield of HEWL species formed during the aggregation process. The values of the Trp fluorescence quantum yield, given by the area under the emission spectra shown in the Fig 7, are the mean of three independent measurements, each performed in quadruplicate. The line of best fit through the data points was obtained by fitting the data with a linear function.

Fig 8 exhibits the time-dependent decrease of the fluorescence quantum yield of Trp during the oligomerization process of HEWL. Analysis of this dependence shows that the Trp fluorescence-monitored kinetics of HEWL aggregation consists of three phases indicative of different types of quenching interactions. Indeed, the Trp fluorescence quenching is most pronounced during the third phase, moderate during the first phase and lower in the second phase (51%, 36% and 13% of the total variation in the fluorescence quantum yield, respectively). Moreover, for all the phases, the Trp quantum yield decreases in a monotonically linear manner with the incubation time. Linear regression analysis of these data indicates that the temporal change of the Trp photophysical features occurs in the third phase with a rate constant (k_3_ = 0.35 h^-1^) that is 2- and 5-fold faster than in the first phase (k_1_ = 0.16 h^-1^) and the second phase (k_2_ = 0.07 h^-1^), respectively. Regardless of the exact nature of these distinguishing spectroscopic features, the Trp fluorescence-monitored kinetics of HEWL amyloid formation displays characteristics (shape of curve and kinetic parameters) quite different from those obtained by the ThT fluorescence-monitored kinetics (Fig 1).

### HEWL aggregation followed by the quenching of tryptophan fluorescence

Fluorescence quenching of Trp residues by acrylamide has been often used as a tool to characterize the solvent accessibility of these amino acid residues in proteins and the polarity of their local environment [59,60]. Since acrylamide is a small and neutral compound able to quench the fluorescence of exposed and buried Trp residues principally via collisional mechanism [59,60], the changes in the Trp environment of HEWL species were probed via the quenching of their fluorescence by this quencher.

**Fig 9:**
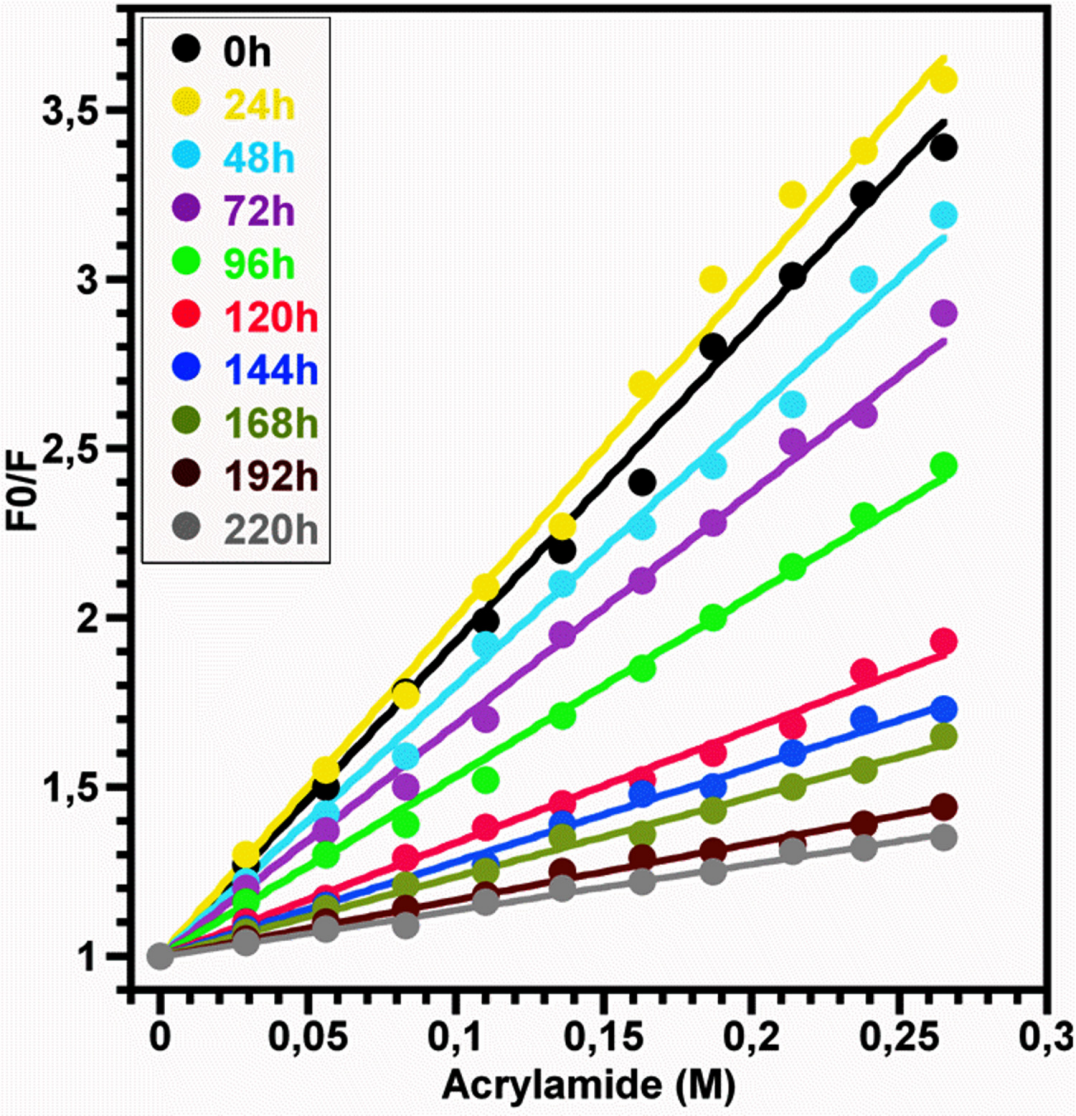
Acrylamide quenching of Trp fluorescence of HEWL oligomers. The data, analyzed according to Stern-Volmer equation [59,60] at each incubation time, represent the variation of the ratio of the Trp fluorescence quantum yield in the absence of acrylamide (F0) to the Trp fluorescence quantum yield at the given quencher concentration (F) as function of the acrylamide concentration. The values of the ratio (F0/F) are the mean of three measurements, each performed in quadruplicate. Measurements were performed for a protein concentration around 10.0 μM.

From the representative concentration-dependent profile for acrylamide quenching of HEWL assemblies (data not shown), the variation of the fluorescence quantum yield of Trp against the quencher concentration was analyzed according to the Stern-Volmer equation [59,60] at each incubation time (Fig 9). The analysis of the Stern-Volmer plot, obtained for the monomeric HEWL, yields a quenching constant Ksv close to 10.0 M^-1^ in acidic pH. During the time course of HEWL aggregation, the quenching constant Ksv decreased to reach a value above 1.0 M^-1^ for the fibrillar aggregates of HEWL. These values, situated within the value range of Ksv obtained for the proteins [59,60], indicate that the oligomerization of HEWL produces a substantial decrease in the accessibility of Trp residues to the acrylamide.

**Fig 10:**
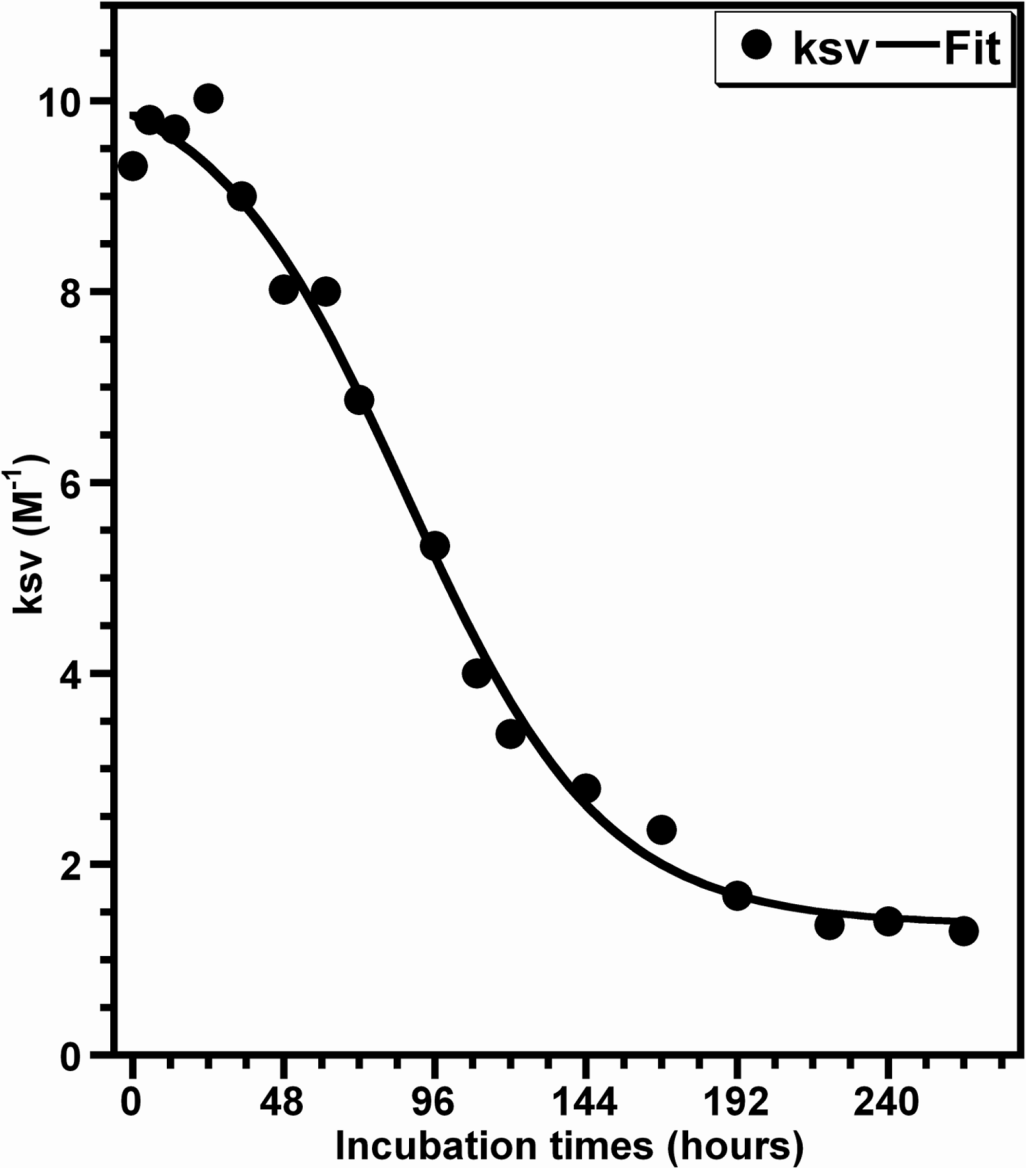
Oligomerization kinetics of HEWL revealed by the fluorescence quenching of Trp. The data represents the temporal evolution of the Stern-Volmer constant (Ksv) of HEWL species formed during the aggregation process. The values of Ksv were deduced from the Stern-Volmer plots shown in the Fig 9. The line of best fit through the experimental data points was obtained by fitting the data with a sigmoidal function [45].

Fig 10 displays the variation of the Stern-Volmer constant Ksv against the incubation time. The analysis of this time-dependence shows that the experimental data are well fitted by a sigmoid curve as shown for those obtained with the ThT fluorescence (Fig 1). Besides the fact that this sigmoid shaped kinetics is indicative of a correlation between the polarity of the Trp microenvironment and the formation of HEWL amyloids, the values obtained for the kinetic parameters (t_1/2_ = 85.9 h, k = 0.034 h^-1^) are quite different from those obtained by the ThT fluorescence-monitored kinetics. In particular, the value of the lag time (t_lag_[Ksv] = 26.7 h) is highly lower than that obtained from the ThT fluorescence curve and this diminution is due essentially to the decrease in the time required to reach the half maximum of the Stern-Volmer constant Ksv (t_1/2_).

### Composition of HEWL aggregates analyzed by reverse phase (RP)-highly performance liquid chromatography (HPLC)

Since several reports have previously observed a partial hydrolysis of lysozyme for long incubation periods in acidic pH [27,29,30,40,52], HEWL samples at different incubation times were subjected to RP-HPLC analysis with the aim to detect the fragments derived from the acid-mediated cleavage of the protein.

**Fig 11:**
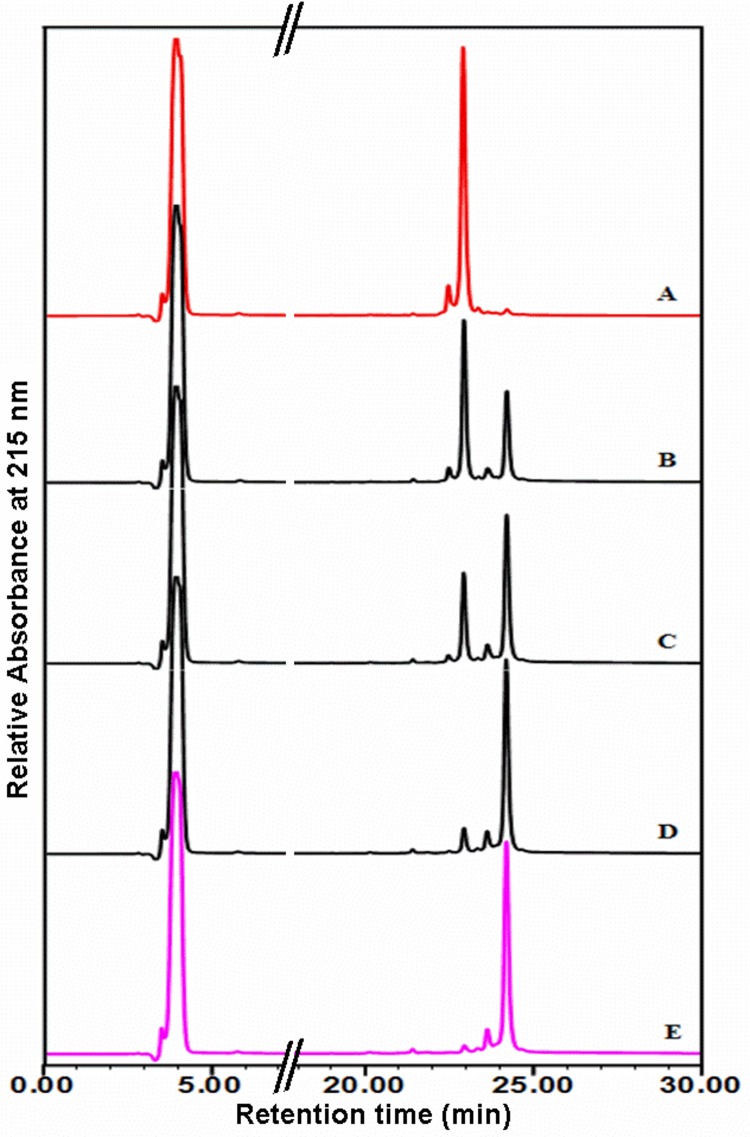
Composition of HEWL aggregates analyzed by RP-HPLC. The panels represent the RP-HPLC chromatograms of HEWL species dissolved in 5 M Gdn-HCl under both non-reducing (-TCEP) and reducing (+TCEP) conditions. The panel A represents the RP-HPLC profile of HEWL species (monomeric or oligomeric) under non-reducing conditions. The panels B-E represent the RP-HPLC profile of HEWL species (monomeric or oligomeric) incubated with the reducing agent TCEP during 1 h, 2 h, 4 h and 6 h, respectively. The constituents of HEWL solutions (protein and peptide fragments) were monitored by UV absorbance at 215 nm.

Fig 11 displays the panels representing the RP-HPLC chromatograms of HEWL samples under both non-reducing (-TCEP) and reducing (+TCEP) conditions. As shown, the chromatogram of the monomeric HEWL without TCEP (Fig 11A) exhibits in Gdn HCl (5 M) one resolved peak (retention time of 22.9 min) together with some minor components contained in the initial stock solution of lysozyme. Interestingly, when the sample of the heat-treated HEWL during 264 h was subjected to the analysis by HPLC, the same chromatogram was obtained, suggesting that HEWL is relatively resistant to the acid hydrolysis during the formation of amyloid fibrils.

However, because HEWL bears four disulfide bonds [24], the HPLC peak, observed at 22.9 min, could contain also the nicked full-length protein. To address this issue, the HEWL samples (monomeric and fibrillar) were analyzed under reducing conditions (+TCEP). As indicated in the Fig 11 (panels B-E), the intensity of the HPLC peak observed at 22.9 min decreases with the incubation time of the protein in the presence of the reducing agent for the benefit of another peak located at 24.2 min. This latter peak, for which the retention time and the intensity remain unchanged for long incubation times (>12 h), corresponds to the full-length HEWL with reduced disulfide bonds. Accordingly, the data in the panels A (disulfide-intact full-length HEWL) and E (fully reduced full-length HEWL) indicate that the incubation of lysozyme in acidic pH at 55°C with agitation generates amyloid fibrils containing exclusively the full-length protein.

## Discussion

In appropriate conditions, many proteins are generally able to form amyloid fibrils [12–43] morphologically similar to those observed in many neurodegenerative diseases as well as localised and systemic amyloidoses [1–5]. In this report, we exploited various structural probes in tandem to characterize the *in vitro* fibrillation of HEWL with the aim to provide details on the physicochemical properties of HEWL species formed during the aggregation process.

Under elevated temperature (~55°C) and acidic pH (~2.0) with agitation (~700 rpm), the ThT fluorescence-monitored kinetics of HEWL aggregation exhibits a sigmoid curve (Fig 1) characteristic of a nucleation-dependent polymerization process as observed for the most amyloid proteins [12–41] including human, hen and equine lysozyme [35,38,52]. Moreover, the AFM pictures (Fig 2) show that, after 10 days of incubation, fibrils with typical amyloid morphology are present in the HEWL sample as observed for other amyloid systems [12–43]. Interestingly, these amyloid fibrils of HEWL exhibit various lengths as observed for insulin [61], α-synuclein [62], the repeat domain of Pmel17 [63] or the peptide Aβ42 [64]. This observed polymorphism for HEWL may result from the formation of multiple oligomeric intermediates during the nucleation phase and/or of different suprabrillar structures. It should be noted that the morphology of the fibrils and the degree of their heterogeneity depend not only on the amino acid sequence of proteins but also of many other factors [19,20,61–66] such as those used in our report (*e*.*g*.: high protein concentration, acidic pH, elevated temperature and agitation). Since these observations are indicative of the formation of HEWL fibrils, the ThT fluorescence analysis of HEWL aggregation has been complemented by other methods in order to provide details on the structural and conformational features of HEWL species formed during the aggregation process.

When monitored by DLS, the kinetics of HEWL aggregation displays also a sigmoidal curve (Fig 4) as observed for the kinetics of HEWL aggregation followed by the ThT fluorescence (Fig 1). However, the DLS-monitored kinetics of HEWL aggregation is characterized by a faster rate for the growth of oligomeric species (k = 0.039 h^-1^) and a longer lag time for the formation of stable nuclei (t_lag_ = 86.7 h) compared to those deduced from the ThT fluorescence-monitored kinetics of HEWL aggregation (k = 0.028 h^-1^ and t_lag_ = 66.6 h). Interestingly, such differences between the kinetic parameters were also observed for the insulin fibrillation [67]. Given that DLS measures the total aggregation of proteins whereas the ThT fluorescence quantifies predominantly the amyloid-like aggregation process, the differences between the two lag-times (Δt = t_lag_[DLS]—t_lag_[ThT]) can be explained by the coexistence of non-fibrillar oligomers with a low percentage of large amyloid fibrils. This hypothesis is supported by the Fig 3C and 3D (representing approximately t_lag_[ThT] and t_lag_[DLS], respectively) which exhibit two distinct peaks corresponding to two populations of HEWL species. Thanks to the intrinsically different sensitivity of these techniques, the major particle population, corresponding to the non-fibrillar oligomers and detected essentially by DLS, appears as possible intermediate species involved in the transition between the native protein and fibrillar oligomers of HEWL. Several reports have shown that the formation of amyloid fibrils by numerous proteins proceeds via multiple and distinct pathways [12,16,19,20,37,40,67–70]. For instance, it was shown that the self-assembly of HEWL in pH 2.0 at 50°C involves two non-competing kinetics pathways depending on the ionic strength of the solution [37,40]: the monomeric fibril assembly (1^st^ pathway) and the oligomeric fibril assembly (2^nd^ pathway) occurring in low- and moderate-salt conditions, respectively. While these observations suggest that the formation of amyloid fibrils by HEWL in our aggregation conditions involves the 1^st^ pathway, the temporal evolution of both the particle size distribution (Fig 3) and the average hydrodynamic radius (Fig 4) of HEWL species exhibit features quite different from those obtained in these reports for the monomeric assembly pathway. The observation of such differences is not surprising because it was previously demonstrated that the aggregation mechanism of proteins differ deeply under agitated or quiescent conditions [31,64,70–74].

In agreement with the data obtained by other groups [38,52,53], our report shows that the oligomeric/fibrillar species of HEWL contain a significant amount of β-sheet structures (Fig 5). However, the temporal evolution of the β-sheet content (Fig 6) does not exhibit a sigmoidal profile and does not occur as a gradual transition. Interestingly, this temporal variation of the β-sheet content differs from that observed for certain amyloid proteins even though there are some similarities [12,16,67,75,76]. For example, the temporal evolution of the β-sheet content, observed for the protein insulin, exhibits a sigmoidal profile as that of the ThT fluorescence curve [67]. For the protein α-synuclein, the amount of β-structures for the pellet samples displays an increase parallel to the ThT fluorescence growth whereas the percentage of β-sheet for the supernatant samples increases to reach a maximum at the end of the lag phase and then decreases progressively up to the completion of the aggregation process [75]. In the case of the peptide Aβ40, the amount of β-structures increases to reach a maximum before the end of the lag phase and then remains almost constant until the ending of the aggregation process [76]. This large diversity, observed for the temporal evolution of the β-sheet content of these proteins and HEWL, reflects the existence of different types of oligomeric intermediates whose the concentration and the structural rearrangement depend strongly on the aggregation conditions [18,40,41,65,67,75–80] and particularly the mechanical agitation [31,64,70–74]. In this regard, between the lag times of the ThT and DLS curves, we observed by DLS the presence of two populations of HEWL species corresponding to non-fibrillar and fibrillar oligomers (Fig 3C and 3D) and by FTIR an increase in the amount of α-helix structures (Fig 6). Moreover, we also observed the presence of random coil structures between 48 h (15.3%) and 72 h (23.3%) (Fig 6). Taken together, these observations suggest that the oligomeric intermediates, involved in the transition between the native protein and β-sheet-rich fibrils of HEWL, are a mixture of unordered, helical, and intermolecular non-fibrillar β-structures.

Given that HEWL is a multitryptophan-containing protein [55,56], the fluorescence characteristics of its Trp residues were used to probe the changes occurring in the tertiary conformation of oligomeric species formed during the protein aggregation process. The Trp fluorescence-monitored kinetics of HEWL aggregation (Fig 8) exhibits distinct monotonic time decreases in the fluorescence quantum yield of the dominating fluorophores Trp^62^ and Trp^108^ with a very slight red shift in their λ_max_ (Fig 7). Given that the solvent-exposed Trp residues in the proteins usually exhibit a decrease in the fluorescence quantum yield with a substantial red shift in λ_max_ [44,57], the behaviour of the photophysical properties of Trp, observed during the aggregation process of HEWL, is due to the quenching of their fluorescence emission. This results from the interactions between the monomeric subunits of oligomeric/fibrillar species which may place quencher groups in the vicinity of these Trp residues [59,60] and/or favour the formation of Trp-Trp stacking [81]. Interestingly, the Trp fluorescence-monitored kinetics of the aggregation of the human and bovine serum albumin exhibits the same profile but the decrease of the Trp fluorescence intensity I_max_ occurs with a fast rate constant [16,77]. By contrast, the Trp fluorescence-monitored kinetics of the aggregation of some amyloid proteins [63,75–78,82] exhibits distinguishing features (shape of curves and kinetic parameters). For example, a close correlation was observed between the amyloid formation kinetics of the protein Pmel17 and the changes in the spectral properties of Trp^423^ (λ_max_ and I_max_) [63]. In the case of the mutant F19W Aβ40, λ_max_ of Trp^19^ undergoes a noticeable blue shift which is temporally correlated with the ThT binding [82]. The differences, observed between these reports, were expected because generally the changes in the photophysical features of Trp (I_max_ and/or λ_max_) for the most proteins are highly site dependent [57]. Accordingly, our results demonstrate that the two fluorophores Trp^62^ and Trp^108^, responsible for ~80% of the total emission of HEWL [55,56], appear to be effective probes of all conformational events occurring during the entire fibrillation process of HEWL and provided kinetic information that is not available from other measurements. It should be noted that the residue Trp^62^, situated in the active site of HEWL [55,56], was shown to be involved in the protein self-association [39,83].

Besides the observations deduced from the data in the Figs 7 and 8, the changes in the solvent accessibility of Trp^62^ and Trp^108^ and the polarity of their microenvironment, during the protein aggregation process, were monitored by examining their fluorescence quenching by acrylamide [59,60]. As seen in the Fig 9, the self-assembly of lysozyme was followed by a robust decrease in the Stern-Volmer constant, indicating that Trp^62^ and Trp^108^, surrounded by a significantly polar environment within the monomeric form (Ksv ≈ 10.0 M^-1^), are transferred in a notably non-polar environment within the fibrillar forms (Ksv ≈ 1.0 M^-1^). This decrease in the polarity of Trp residues was also observed for different peptides and proteins but the variation between the Ksv values of the monomeric and fibrillar species of these proteins is low compared to that obtained in the present report [39,63,84,85]. Since Trp^62^ and Trp^108^ remained partially buried during the time course of fibril growth of HEWL (Fig 7), the decrease in their accessibility to acrylamide may be the consequence of the slower translational and rotational diffusion rates of HEWL species unrelated to any change in the protein conformation and/or may result from geometrical masking factors [54,55,86]. However, generally the reduction in the translational and rotational motilities of Trp residues might account for 50% in the reduction of the Stern-Volmer constant of Trp [86]. Moreover, the temporal evolution of Ksv (Fig 10) follows a sigmoidal profile which is a mirror image of the ThT fluorescence (Fig 1) and <Rh> (Fig 4) curves, indicating that the formation of oligomeric/fibrillar species of HEWL is correlated with the decrease in the polarity of the local environment of Trp residues. This finding was also observed for the mutant Y^39^W of α-synuclein for which the temporal evolution of the fluorescence anisotropy and Ksv mirror each other in an exponential profile [82]. However, this α-synuclein mutant exhibits both a significant blue shift in λ_max_ and a low variation in Ksv. Besides the observation of this sigmoid shaped kinetics (Fig 10), the value of the lag time (t_lag_[Ksv] = 26.7 h) is highly lower than that obtained from the ThT curve. Accordingly, the diverse conformational modifications, responsible for the low polarity of the local environment of the fluorophores Trp^62^ and Trp^108^, appear to be more pronounced during the early folding events occurring in the formation of HEWL amyloids. Interestingly, we observe that more than 50% of the total β-sheet structures were formed during the first 48 hours (Fig 6), indicating that the transition from α-helix to ß-sheet participates highly to the low polarity of the microenvironment of the fluorophores Trp^62^ and Trp^108^.

Numerous lines of evidence have indicated that the incubation of proteins in low pH [52,87,88] results in a significant chemical degradation of proteins at peptide bonds highly susceptible to acidic hydrolysis [89]. When the integrity of HEWL within the oligomeric/fibrillar species was analyzed by RP-HPLC, no product of the acidic hydrolysis was detected under both non-reducing and reducing conditions (Fig 11). This result, indicating that the incubation of lysozyme for several days (~2 weeks) generates amyloid fibrils containing exclusively the full-length protein, diverges with previous reports which exhibit major [27,30] or minor [29,40,52] hydrolysis of HEWL during the formation of amyloid fibrils. Indeed, Frare *et al*. [27] showed that the incubation of lysozyme for ten days in pH 2.0 at 65°C results into amyloid fibrils that are largely composed of fragments deriving from the protein hydrolysis. Furthermore, Mishra *et al*. [30] not only confirmed these results under non-reducing and reducing conditions but also showed that the acid-mediated cleavage of lysozyme occurs at the initial stage of the incubation (within the first 24 h) and contributes to the formation of amyloid fibrils. In contrast, other reports showed that the formation of fibrils of hen egg white [29,40] or equine lysozyme [52] in pH 2.0 at 57°C was accompanied by some acidic hydrolysis which is more pronounced at a later stage of incubation (1 to 2 weeks). Moreover, they observe that a majority of fibrils contains mostly the full length lysozyme [29,40,52] and the acid-mediated cleavage of lysozyme is not required for the formation of amyloid fibrils [40,52]. These differences, observed at the level of the extent of the acidic hydrolysis of HEWL, the time points where it occurs and its influence on the course of the aggregation process, are probably due to the type of techniques and/or the conditions (with or without reducing agents) used by each group to analyze the molecular composition of HEWL species. Conversely, the observation in our study of the intact lysozyme within the oligomeric/fibrillar species results from the use of an agitation of 700 rpm which may prevent the acidic hydrolysis of the monomeric protein during a prolonged heating by favouring the stability of oligomeric intermediates and/or by enhancing the *in vitro* growth of HEWL fibrils.

## Conclusions

In summary, this work illustrates the advantage to use multiple and complementary spectroscopic methods for exploring the structural features of oligomeric/fibrillar species formed under controlled conditions during the entire aggregation pathway of hen egg-white lysozyme. Indeed, the study of HEWL self-assembly under our aggregation conditions highlights the integrity of the full-length lysozyme within the oligomeric/fibrillar species (HPLC) and reveals multiple and kinetically distinct steps such as the conformational changes (quantum yield and quenching of Trp fluorescence and FTIR spectra) and the size growth (DLS) followed by the fibril formation (ThT binding and AFM). Accordingly, this experimental approach appears to be of general applicability to investigate the aggregation of other physiologically important proteins and the efficacy of compounds that disrupt or alter the aggregation of those amyloid proteins. Moreover, this work provides further insight into the nature of factors that affect the process of aggregation of HEWL. Hence, despite the similarities between the fibrillar structures formed from the proteins, a detailed approach is needed to establish the mechanisms underlying oligomerization, the formation of oligomeric intermediates, and the formation, association, and growth of fibrils for every individual protein and in each aggregation condition. Finally, given that the structural features (local, secondary, oligomeric and fibrillar structures) observed for the agitation-induced fibril formation of HEWL have not been reported before for this amyloid system, we believe that the mechanistic insights gained from the present work would be useful in the design of anti-amyloid therapeutics by targeting various stages of the process of aggregation proteins.
